# The Distribution of Lectins across the Phylum Nematoda: A Genome-Wide Search

**DOI:** 10.3390/ijms18010091

**Published:** 2017-01-04

**Authors:** Lander Bauters, Diana Naalden, Godelieve Gheysen

**Affiliations:** Department of Molecular Biotechnology, Faculty of Bioscience Engineering, Ghent University, Coupure links 653, 9000 Ghent, Belgium; lander.bauters@ugent.be (L.B.); diana.naalden@ugent.be (D.N.)

**Keywords:** nematode, C-type lectin, hevein, bioinformatic analysis

## Abstract

Nematodes are a very diverse phylum that has adapted to nearly every ecosystem. They have developed specialized lifestyles, dividing the phylum into free-living, animal, and plant parasitic species. Their sheer abundance in numbers and presence in nearly every ecosystem make them the most prevalent animals on earth. In this research nematode-specific profiles were designed to retrieve predicted lectin-like domains from the sequence data of nematode genomes and transcriptomes. Lectins are carbohydrate-binding proteins that play numerous roles inside and outside the cell depending on their sugar specificity and associated protein domains. The sugar-binding properties of the retrieved lectin-like proteins were predicted in silico. Although most research has focused on C-type lectin-like, galectin-like, and calreticulin-like proteins in nematodes, we show that the lectin-like repertoire in nematodes is far more diverse. We focused on C-type lectins, which are abundantly present in all investigated nematode species, but seem to be far more abundant in free-living species. Although C-type lectin-like proteins are omnipresent in nematodes, we have shown that only a small part possesses the residues that are thought to be essential for carbohydrate binding. Curiously, hevein, a typical plant lectin domain not reported in animals before, was found in some nematode species.

## 1. Introduction

Lectins occur in plants, animals, and microorganisms. Although they are best known for their carbohydrate binding properties, some of them are also involved in protein–protein, protein–lipid, or protein–nucleic acid interactions [1]. Lectins are defined as proteins that have at least one non-catalytic domain that reversibly binds to a specific mono- or oligosaccharide [2]. These carbohydrate-binding proteins may be involved in a variety of functions, ranging from innate immunity to glycoprotein synthesis [3].

The first lectin was isolated from seeds of the castor bean, *Ricinus communis*, from which its name was derived: ricin. Stillmark [4] discovered that this extremely toxic protein was able to clot red blood cells. It took over 60 years to assign the agglutination activity to the ability of lectins to specifically recognize and bind carbohydrate structures on the surface of erythrocytes [5]. Since that initial find, more lectins have been reported and characterized in other plant species, and their omnipresence in the plant kingdom is supported by several genome and transcriptome analyses [6,7]. It took almost a century after the initial discovery of lectins in plants to report lectins in animals. A hepatic lectin was found in the rabbit liver in 1974 and was claimed to be the first lectin of mammalian origin [8]. However, it seems likely that the first animal lectins were revealed earlier than plant lectins since the first lectin activity was recorded by Flexner and Noguchi in 1902, but it was just not identified as a carbohydrate-binding protein [9]. Although plant and animal lectins both bind carbohydrates, the family is very diverse, revealing a wide variety of lectin folds and specific carbohydrate-binding sites [10]. The structural diversity of the carbohydrate-binding domain, rather than carbohydrate specificity, is used to divide the lectin group into several protein families.

Animal lectins were initially classified into two major distinct classes: the C-type (Ca^2+^-dependent) and the S-type (thiol-dependent, known as galectins) lectins [11]. Over the years it became clear that the lectin group is far more diverse than initially stated. At least 12 structural families are present in animals, although not all of them are well characterized [1,12]. Plant lectins are grouped into 12 different families according to their carbohydrate-binding domains [6]. Despite their diversity, plant and animal lectins have some carbohydrate recognition domains in common, providing evidence for a common ancestor. Two examples are the legume and ricin-type lectins from plants, which are also found in animals, where they are designated as L-type and R-type lectins, respectively [13].

Although research regarding lectins in nematodes is scarce, several reports of nematode lectins have been made previously. Most research has focused on the model organism *Caenorhabditis elegans*, but lectins have been described in other nematodes as well. Thus far, only the two biggest animal lectin families have been reported in nematodes: C-type lectins and galectins [14,15,16,17]. In this research we give an overview of the number and type of lectins present in nematodes, with a focus on C-type lectins. To our knowledge, this is the first time an attempt has been made to extract the different types of lectins from several nematode genomes. Drickamer et al. [18] already gave an overview of the different potential C-type lectins present in the genome of *Caenorhabditis elegans*, leading to the discovery of 125 proteins with one or more C-type lectin-like domains (CTLDs), grouped in nine different categories. Another study used updated techniques and found 278 proteins in *C. elegans* containing at least one CTLD [19]. Recently, Pees et al. [20] reviewed the presence and potential function of C-type lectin-like domains in invertebrates, thereby making a new classification of CTLD-containing sequences in *C. elegans*.

The availability of several nematode genomes gave us the ability to perform a thorough search across different species regarding lectins. This search shows that CTLDs are the most abundant type of lectin domain in all nematode species. In addition, the number of proteins containing a CTLD is much higher in free-living, soil-dwelling nematodes compared to species with different lifestyles, except for Steinernema species. Hevein, a typical chitin-binding plant lectin, was retrieved from several nematode genomes in this study. To our knowledge, this is the first report of the presence of a hevein-like domain in animals. The carbohydrate-binding properties of each type of lectin were investigated in silico, but still need to be confirmed experimentally. The lectin-like proteins identified in this research should be considered as potential lectins until carbohydrate binding is supported by experimental evidence. The results reported in this work give a better overview of the distribution of lectin-like domains in the phylum Nematoda.

## 2. Results

### 2.1. Selection of Lectin-Like Domains

No specific nematode-lectin domain profiles exist to date. Most lectins in nematode data are not annotated as such, so a more indirect approach was used to extract lectin domains from nematode sequence data. Several nematode genomes are available, but only a few nematode genomes were used for a thorough analysis in this study. One or more well-characterized genomes were selected, representing nematode groups with different lifestyles. The genome of *C. elegans* was chosen as a representative for nematodes with a free-living lifestyle, *Ascaris suum* and *Brugia malayi* represent the group of animal parasitic nematodes, while *Globodera pallida*, *Meloidogyne incognita*, *Meloidogyne hapla*, and *Bursaphelenchus xylophilus* represent the plant parasitic nematodes. Available Pfam profiles of plant lectin and animal lectin domains were used to query several nematode genome databases. Since not all Pfam profiles exclusively represent a lectin domain, all hits were checked manually before withholding them. All hits were blasted against Swissprot. If the top hit did not contain any annotation regarding (potential) carbohydrate-binding activity, the sequence was discarded. Due to the fact that some profiles were not specific for some lectin-like domains, two types of lectin domains were checked in more detail before annotating them as potential lectins: I-type lectins (PF07686) and M-type lectins (PF01532).

M-type lectins are closely related to mannosidases found in the endoplasmic reticulum, but they lack mannosidase activity due to the absence of some key residues and an important disulfide bond [21,22]. A phylogenetic tree containing all potential M-type lectins was constructed using the maximum likelihood method (results not shown). Sequences lacking the two cysteine residues needed for the important disulfide bond or sequences lacking a key glutamic acid (Glu) residue needed for mannosidase activity clustered together in one branch of the tree. A total of 35 sequences lacking key residues needed for mannosidase activity clustered together in this branch and were considered potential M-type lectins.

I-type lectins owe their name to the presence of the immunoglobulin-like domain in their structure that mediates carbohydrate recognition. Siglecs are the major subfamily of I-type lectins and are characterized by a single N-terminal V-set domain (PF07686) and a single transmembrane domain [23]. V-set domains are common in diverse protein families, like immunoglobulins and T-cell receptors. Hence, a detailed analysis was needed to extract potential I-type lectins from all the hits obtained by a profile hidden Markov model analysis (or HMMer-analysis) using the V-set profile. Potential I-type lectins had to meet three criteria [23]: (1) contain one or two N-terminal V-set domains; (2) have a single transmembrane domain; and (3) have a V-set domain containing an odd number of cysteine residues (typically 3). None of the initially found sequences containing a V-set domain met all of these criteria. These results are in agreement with previous findings that I-type lectins are confined to vertebrates [24].

After a careful selection of all potential lectin-like proteins, lectin-like domains were extracted from the nematode sequences to design a new HMM profile for each type of lectin. The new profiles were used to query the nematode databases a second time. In addition to the genome data, transcriptome data of six plant parasitic nematodes were searched as well. New hits were checked manually, as previously described. Results originating from the transcriptome data should be considered with caution since these datasets only present a partial image and are not as reliable as genome data. Six plant-type lectin domains were not present in any of the studied nematode species: *Agaricus bisporus* agglutinins, Amaranthins, Cyanovirins, *Galanthus nivalis* agglutinins, Jacalins, and Nictaba lectin. Three animal lectins were not reported as well: Intelectins, P- and I-type lectins. P-type lectins are also known as mannose-6-phosphate receptors, recognize phosphorylated mannose, and play a role in the generation of functional lysozomes. P-type lectins are conserved in vertebrates but seem to be absent in invertebrates [24]. Sequences with some similarity to the P-type lectins found in invertebrates lack most of the residues found in the sugar-binding sites. Hence, these sequences are not classified as P-type lectins [12]. Intelectins are lectins with a highly conserved protein sequence involved in early embryogenesis, host–pathogen interactions, and iron metabolism. Intelectins are absent from invertebrates, although they have been found in lancelets (*Branchiostoma lanceolatum*), which are considered the transition state between vertebrates and invertebrates [25,26]. None of the bacterial lectins was present in any of the queried nematode species. F-type lectin domains, or fucolectins, have been retrieved from bacteria, vertebrates, and invertebrates, but so far they have not been reported in the phylum Nematoda [27]. In this study we identified a sequence with a possible F-type lectin domain in *A. suum* and *C. elegans*. In *C. elegans*, the F-type lectin-like domain is coupled to a C-type lectin-like domain.

Results of the second round of profile searching are summarized in Table 1. Lectin domains that are not present in any of the nematode species are not shown in this table. The numbers in this table reflect the general idea that most animal lectins belong to two families: the C-type lectins and the galectins [11]. Looking at CTLDs, *C. elegans* has on average eight times more genes containing this domain compared to other nematode genomes. In addition, *C. elegans* is the only nematode of the seven investigated genomes that has genes containing hevein-like domains.

### 2.2. Predicting Carbohydrate Binding Properties

Although the identified domains are similar to previously characterized lectin domains, it does not guarantee carbohydrate binding. Identifying potential lectins can be considered a two-step process in which lectin-like sequences are identified by a profile analysis, after which candidates are screened for the necessary sugar-binding residues [12]. Numbers in Table 1 give an indication of the prevalence of several lectin-like domains in nematodes, but their carbohydrate binding activity still needs to be investigated. In the following paragraphs we will further investigate the carbohydrate-binding properties of potential lectin domains retrieved from the nematode genome data and make predictions about their carbohydrate-binding abilities.

L-fucose binding of the F-type lectin domain was assigned to a specific motif: HX(26)RXDX(4)R/K [28]. Previous studies have shown that mutating one residue might be sufficient to abrogate the carbohydrate-binding properties of the domain [29]. The two identified F-type lectin-like domains in *C. elegans* and *A. suum* do not possess the consensus fucose-binding motif. Bishnoi et al. [27] showed that several variations are possible in this motif, but their carbohydrate-binding capabilities have not yet been investigated. Further research is necessary to annotate the two F-type lectin-like domains as true lectins.

Calnexins/calreticulins interact with carbohydrates through six specific amino acids in their sequence: two tyrosines (Tyr), a lysine (Lys), a methionine (Met) and two glutamates/aspartates (Glu/Asp) [30,31]. Both calnexin and calreticulin are important lectins aiding the process of protein folding in the endoplasmic reticulum. All potential calnexins/calreticulins were aligned with ClustalO to study the conservation of these important residues. Fifteen out of 24 sequences contained all residues that were shown to be essential for carbohydrate binding, hence these proteins might be considered true lectins (Supplementary Figure S1).

The carbohydrate-binding domain in legume lectins is formed by four loops in the tertiary structure of the protein. Legume lectins display a large divergence in their carbohydrate specificity, and hence the residues in the carbohydrate binding domain are very variable. L-type lectins are involved in protein sorting in animals; their role in plants is not completely clear but they might be involved in immunity [32]. Despite the large variability, a few key amino acid residues are largely conserved among these lectins: an aspartate in the first loop, a glycine in the second loop, and an asparagine combined with an aromatic amino acid in the third loop [33,34]. In addition, a histidine residue was shown to be essential for carbohydrate binding [35]. Potential legume lectins were aligned and scanned for the presence of key residues. Ten sequences contained all of the described residues. Except for a protein originating from *A. suum* and *G. pallida*, the remaining sequences contained all but one of the key residues (Supplementary Figure S2). Although experimental testing is necessary, results indicate that 13 out of 15 proteins are likely true carbohydrate-binding legume lectins.

Ricin-B-type lectin (R-type) domains contain three tandemly repeated subdomains, designated as α, β, and γ. Each subdomain has a characteristic aspartate (D) residue and a glutamine-x-tryptophan (QXW) motif that might be involved in carbohydrate binding. Previous mutation studies have shown that these three residues occur in each subdomain and are involved in carbohydrate binding [36,37,38]. Experimental data suggested that a single complete binding triad is sufficient to bind a carbohydrate structure [39]. All potential Ricin-B-type lectin domains identified by a profile analysis were aligned and sequences having at least one full binding triad were considered as carbohydrate-binding. Forty-seven out of 69 sequences met this criterion (Supplementary Figure S3). Since Ricin-B lectin domains with substitutions in the triad might also bind carbohydrates, these results should be considered with caution. For instance, the tryptophan might be replaced by phenylalanine without changes in protein function [37,39].

So far, the data regarding the identification of key residues in carbohydrate binding of LysM domains are scarce. Mutagenesis studies showed that a key aromatic amino acid is needed for successful carbohydrate binding in a eukaryotic LysM domain [40]. In addition, a recent study determined the solution structure of a bacterial LysM domain to identify residues involved in interaction with carbohydrate molecules [41]. To our knowledge, no similar attempts have been made for eukaryotic LysM domains. Upon alignment of the nematode-derived LysM domains, a clear separation into two groups was seen: a group with three conserved cysteine residues and a group without these residues (Supplementary Figure S4). It was suggested before that the cysteine residues might form disulfide bonds aiding in stabilizing a eukaryotic LysM domain [40]. The prominent cysteine residues are absent in the Pfam profile (PF01476), which is predominantly constructed with bacterial sequences. The key aromatic amino acid described by Ohnuma and colleagues [40] is only present in the cysteine-rich sequences together with one (T13) of the described key residues in bacteria [41]. The sequences lacking conserved cysteine residues contained some (T13, L14, and F40) of the described bacterial key residues (Supplementary Figure S4). It is difficult to predict carbohydrate-binding properties from these results; hence, only experimental data can give a decisive answer.

Sequences with similarity to M-type lectins have gone through a thorough analysis, as described above. An alignment shows the potential M-type lectins in nematodes, indicating the residues discriminating them from mannosidases (Supplementary Figure S5).

A total of 76 sequences with similarity to class V chitinases were identified in the queried nematode genomes. To annotate them as potential lectins, they should not have any catalytic activity. Three acidic residues, grouped in the DxDxE motif in the catalytic site, are responsible for hydrolyzing chitin. Chitinase-like proteins lacking catalytic activity are known to have at least one non-conservative mutation in the key acidic residues [42]. Twenty-five sequences possess all three key residues, ruling them out as potential chitinase-like lectins (Supplementary Figure S6). Although the remaining 51 sequences are most probably devoid of any chitin-hydrolyzing activities, their chitin-binding properties should still be confirmed.

A large group of potential galectins (galactoside-binding lectins) was found in the nematode genome data. A total of 237 sequences contained one or more galectin-like domains. All galectin-like domains (366 in total) were searched for a consensus carbohydrate-binding domain, confirming their lectin characteristics. The eight residues thought to be important in carbohydrate binding, in vertebrate as well as invertebrate organisms, are His44, Asn46, Arg48, Val59, Asn61, Trp68, Glu71, and Arg73 (the numbers correspond to those of human galectin-1) [43]. An alignment of the galectin-like domains was searched for these residues and their described variants in *C. elegans* [43]. A total of 74 galectin-like domains contained the conserved residues; these domains were present in 57 individual protein sequences. This number is probably a large underestimation. Mutation analyses on the eight key residues showed that not all of them are essential for carbohydrate binding, although they can influence sugar specificity. The alignment of the 74 selected domains can be seen in Supplementary Figure S7.

Not all proteins containing a C-type lectin-like fold have carbohydrate-binding properties; a large subgroup is known to bind protein ligands instead, whether or not they are Ca^2+^-dependent. Carbohydrate-binding properties have been associated with five key amino acid residues. These five residues (Glu185, Asn187, Glu193, Asn205, and Asp206; the numbers correspond to rat mannose-binding protein) are part of a Ca^2+^ binding site in the C-type fold [44]. The first two residues are known to be involved in the specificity of sugar binding. Mutating these residues to Gln and Asp, respectively, switches the binding specificity from mannose/glucose to galactose [45], although variations are possible [46,47]. All C-type lectin-like domains were searched for the presence of key residues. If three out of five residues were present, the domains were considered as carbohydrate binding. This approach was also used in previous studies [18,48]. Seventy-seven domains met the criteria, corresponding to 75 CTLD-containing sequences (Supplementary Figure S8).

Proteins with a hevein-like domain are discussed in detail in the following section.

### 2.3. Hevein-Type Lectins

**Figure 1:**
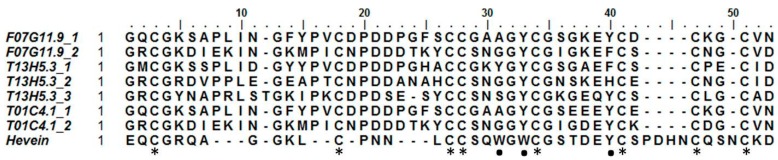
Alignment of the eight discovered hevein domains in *Caenorhabditis elegans* with the original hevein peptide from *Hevea brasiliensis*. Asterisks indicate the eight conserved cysteine residues forming four disulfide bridges; dots show the aromatic amino acids and serine residue involved in chitin binding.

Hevein was discovered in the rubber tree (*Hevea brasiliensis*), where it is involved in the coagulation of latex. Hevein-like peptides bind to chitin, a polymer of *N*-acetylglucosamine, in a calcium-dependent manner [49]. These 29–43 amino acid chitin-binding domains are rich in cysteine, glycine, and several aromatic amino acids. Based upon the number of cysteine residues, they can be classified into three groups: 6C-, 8C-, and 10C hevein-like peptides [50]. To our knowledge, no such domains have been reported outside of the kingdoms of plants and fungi [6]. Three sequences, originating from *C. elegans*, appear to contain a domain similar to a hevein domain. Two of them (T01C4.1 and F07G11.9) contain two hevein-like domains, while T13H5.3 has three (Figure 1). To exclude any possible contamination, additional available genomes of other nematode species were queried for this domain (see Table S1). Several species seem to have genes containing one to three hevein-like domains. In particular, all free-living nematode species investigated in this study possess such genes. In addition, hevein-like domains were also detected in some insect and animal parasitic nematodes assigned to clade 8a, 9b, 10a, and 10b (classification according to [51]). No hevein-like domains were found in nematode species from clades 2a, 8b, and 9c. A total of 136 hevein-like domains were found in 32 species of the additional queried genomes. Only nine of these domains did not contain the eight conserved cysteine residues that are present in the original discovered hevein and hevein-like peptides. The eight cysteine residues form four disulfide bridges to stabilize the peptide [52]. Hevein-like peptides show three conserved aromatic amino acid residues, responsible for chitin binding (residues 21 (Trp), 23 (Trp), and 30 (Tyr)) and a conserved serine residue (S19) [53]. Two of the aromatic residues are conserved in all hevein-like domains of *C. elegans*, while at position 21 only T13H5.3_1 shows an aromatic amino acid (Figure 1). All three *C. elegans* proteins containing a hevein-like domain also have a glycoside hydrolase family 18 (GHF18) domain (PF00704) and a predicted N-terminal signal peptide in their sequence (Figure 1). In addition, the two longest proteins both have a stretch of LysM domains preceding the GHF18 domain. In accordance with these three *C. elegans* genes, the hevein containing genes in the additional investigated nematode genomes were also combined with a GHF18 domain and/or LysM domains. Furthermore, 16 sequences contained a domain with homology to the plant self-incompatibility protein S1 domain (PF05938). This domain was initially discovered in Papaver and has only been found in plants and some nematodes [54].

A profile HMM, constructed from the seven hevein-like domains in *C. elegans*, was used to query the UniprotKB database. The search was restricted to Animalia. All nematode hits (46) originated from free-living species or animal parasites, divided between clades 8A, 9, and 10 [51]. In addition, 43 hits originated from Branchiostoma (lancelets) and eight from Arthropoda (including the pea aphid, the red spider mite, the lygus bug, the sea louse, and the salmon louse). These results, together with the results in Table S1, indicate that the hevein-like domain is not restricted to plants and fungi but is also present in animal species.

### 2.4. C-Type Lectin-Like Domains

Remarkably, *C. elegans* has 5- to 10-fold more genes containing at least one CTLD compared to the investigated animal and plant parasitic nematodes. The number of CTLD-containing genes in *C. elegans* found in this research was twice the number predicted by Drickamer et al. [18], but was comparable to the number of CTLD-containing genes predicted by Pees et al. [20]. Next to the seven genomes that were thoroughly scanned in this study for potential lectin-like proteins, another 63 genomes of free-living (11), entomopathogenic (7), and animal parasitic (45) nematodes were queried to look for CTLDs (Table S1). Results showed that not only *Caenorhabditis* species, but also other free-living nematodes (for instance, *Pristionchus* spp.) have a high number of genes containing CTLDs. The only exception was the free-living nematode *Rhabditophanes* sp. KR3021, which only contained 34 genes with a CTLD in its genome. Interestingly, all *Steinernema* species contained high numbers of CTLD-containing genes as well. *Steinernema* species are parasitic nematodes capable of infecting a broad range of insect species. In contrast, *Heterorhabditis bacteriophora*, another entomopathogenic nematode, has a very low number of CTLD-containing genes in its genome. It can be concluded that free-living nematodes and *Steinernema* species have acquired a large collection of genes containing one or multiple CTLDs, while plant and animal parasitic nematodes have much smaller collections. A Pearson’s correlation test (*p*-value = 0.692 > 0.05) showed that this observation could not be attributed to a difference in genome size between different nematode species.

Drickamer et al. [18] organized 125 proteins from *C. elegans* containing a CTLD into nine different families according to the number of CTLDs and the presence of other protein domains. In this research we used the more recent classification by Pees et al. [20], which divides CTLD containing genes into six different classes according to the presence of protein domains. We added one group to this classification for genes containing four CTLDs. This additional group consisted of only a single sequence from *A. suum* (Table 2).

A reciprocal blast was performed to look for homologous CTLD sequences present in all seven nematode species under study. Results showed that three CTLD-containing proteins are present in all investigated species. The first sequence was classified into class I in *C. elegans* (F25B4.9) according to the classification of Pees et al. [20]. This group of sequences only contains a single CTLD. The sequence of *G. pallida* is the only sequence that is not predicted to have a secretion signal and where the CTLD is preceded by a protein kinase domain (PF00069) and followed by a small helix-loop-helix DNA-binding domain (PF00010). The second protein was also classified in class I (F32E10.3), while the other was classified in class VI (F47C12.2) in *C. elegans*. All F32E10.3 homologues have a single CTLD, followed by a large C-terminal domain without any predicted protein domains, except for the sequence of *A. suum*, which only consists of a short N-terminal domain followed by a CTLD. The sequences of the two *Meloidogyne* species and of *A. suum* are the only ones without a predicted N-terminal secretion signal. Although results of the reciprocal blasts with F47C12.2 indicated homology between the sequences, the homologous sequence originating from *B. malayi* was classified in class I instead of class VI. All of the proteins are predicted to have an N-terminal signal peptide, except for the proteins of the two *Meloidogyne* species and *A. suum*.

One of the best known characteristics of CTLDs is the “WIGL” motif, which is highly conserved in CTLDs. The “WIGL” motif is involved in the formation of hydrophobic cores in the tertiary structure of the C-type lectin fold [55]. The tertiary structure is stabilized by two highly conserved disulfide bridges. According to Zelensky and Gready [55], there are five more highly conserved residues next to the cysteines involved in the formation of the two disulfide bridges. Three of them (W, G, and L) are residues from the “WIGL” motif. All nine residues seem to be conserved across different species [55]. Table 3 shows the presence of the nine residues in nematode CTLDs, all of which display a high degree of conservation. Almost half of the investigated sequences contain all four cysteine residues to form both disulfide bridges. About 25% only contain the first (C1) and last (C4) conserved cysteine residue to form the outer disulfide bridge (Table 3).

**Figure 2:**
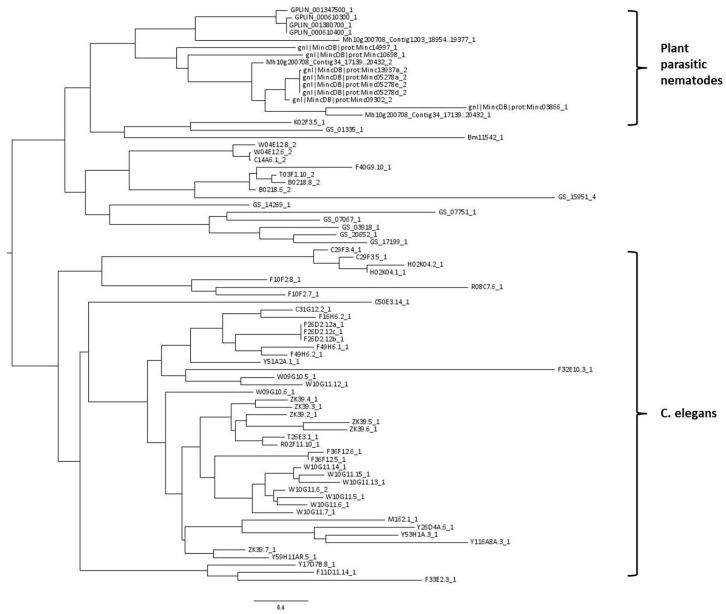
Midpoint rooted phylogenetic tree of 77 nematode C-type lectin-like domains (CTLDs) predicted with the maximum likelihood algorithm. Taxon labels give the full genome accession code of the gene containing the CTLD, followed by a number indicating which CTLD of that particular gene is used in this analysis. Explanation of prefixes: Bm: *Brugia malayi*, GPLIN: *Globodera pallida*, Mh10: *Meloidogyne hapla*, Mincdb: *Meloidogyne incognita*, GS: *Ascaris suum*. Taxon labels without any of the previous prefixes originate from *Caenorhabditis elegans*.

A phylogenetic tree was constructed using all 77 CTLDs predicted to have carbohydrate-binding properties. The tree shows that CTLDs originating from plant-parasitic species cluster together, while there is also a large cluster containing *C. elegans* CTLDs (indicated in Figure 2). The remaining cluster is a mixture of sequences originating from *C. elegans*, *B. malayi*, and *A. suum*.

### 2.5. General Overview

**Figure 3:**
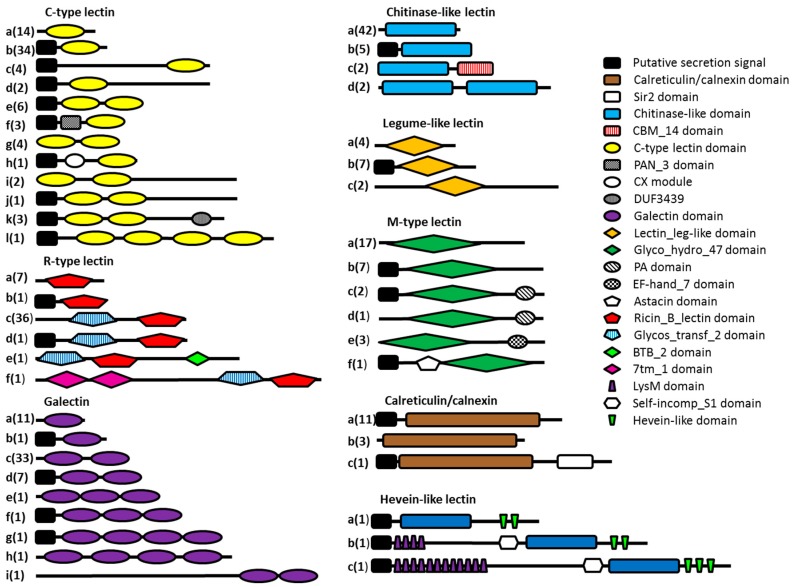
Schematic overview of the different types of potential lectins present in the seven investigated nematode genomes. Eight lectin classes are represented here, each divided into several subclasses (denoted by letters) according to the presence of other predicted protein domains. The number of sequences present in each subclass is indicated in brackets.

All lectin-like protein sequences containing key amino acid residues indicating carbohydrate-binding properties were investigated in more detail. The Pfam database of protein domains was queried to study the domain organization of the potential lectin domain-containing proteins (Figure 3). Thirty-four percent of the lectin domain-containing proteins consist of a lectin domain flanked by regions without a known domain assignment. Only 20% of the protein sequences consist of a lectin domain combined with one or more other protein domains other than a predicted N-terminal secretion signal. Although a minority, some protein domains are interesting and seem to be related to the function of the lectin domain. The combination of a C-type lectin domain combined with a PAN module has been found in nematodes before. The PAN module is a domain found in the N-terminal domains of the plasminogen/hepatocyte growth factor family, the apple domains of the plasma prekallikrein/coagulation factor XI family and in various nematode proteins. The PAN module (also known as the CW domain) is thought to be involved in protein- or carbohydrate-binding interactions [56]. The CX domain, which is combined with a C-type lectin domain, seems to be nematode-specific, but so far no function has been assigned to this domain. R-type lectin domains are preceded by a glycosyltransferase domain in almost all cases. The lectin domain may play an important role here in binding the substrate needed by the glycosyltransferase. Two of the probable chitinase-like lectin domains are followed by carbohydrate-binding module 14 (CBM14), which is known to bind chitin [57]. Three potential M-type lectins have a Protease-Associated (PA) domain. This domain is normally associated with proteases [58], but has been found in combination with α-mannosidases as well, although its function remains elusive [22]. All hevein-like domains are associated with a chitinase domain (GHF18), strengthening the hypothesis of the chitin-binding properties of the hevein-like domain. In addition, two of the hevein domains are combined with a stretch of LysM domains, which are known for their peptidoglycan- and chitin-binding properties [59].

## 3. Discussion

Lectins have been the topic of numerous research projects in plants and animals, but information regarding this topic in nematodes is rather scarce. Lectin research involving nematodes started about 30 years ago, elaborating on the effect of non-nematode-derived lectins binding on the nematode surface [60,61,62]. Since the publication of the genome of *C. elegans* in 1998, a platform was created enabling scientists to investigate nematode lectins [63]. Numerous nematode genomes and transcriptomes have become publicly available since then. Research regarding lectins has mainly focused on C-type lectins [18,64], but other lectins were also investigated in more detail, like galectins [65] and calreticulin [66]. To our knowledge, this is the first attempt to make a summary of the types of lectin-like proteins present in nematodes, using the available nematode genomes. This research showed that the numbers and types of lectin-like proteins in different nematode species are quite diverse and confirmed the earlier statement of Drickamer [11] that most animal lectins belong either to the group of C-type lectins or galectins. The investigated *Heterodera* and *Pratylenchus* species seem to be an exception to this rule, but here we only queried the transcriptome data of these species. Lectin domain-containing genes were retrieved from nematode genomes using an HMM-based search. Not all animal lectin-like domains have a specific HMM-profile (e.g., ficolins, intelectins, F-type lectins, etc.), hence only the most important lectin-like domains were included in this search.

Experimental evidence in *C. elegans* suggests that a significant share of C-type lectin-like proteins is involved in the immune response upon bacterial invasion. Since there are only a few genes that are upregulated by more than two pathogen species, it seems that the action of C-type lectin-like proteins is specific to a certain type of pathogen. It was found that approximately one-fifth of the genes with a CTLD present in the genome of *C. elegans* are induced by pathogen infection (summarized by [19] and [20]). Proteins with a galectin-like domain are involved in immune responses as well, since the expression of galectin-like encoding genes is induced upon pathogen infection in *C. elegans* [67,68]. Next to a role in innate immunity, both CTLDs and galectin-like domains from parasitic species might have a role in suppressing host immune systems. CTLDs have been found in the secretome of several parasitic nematodes and were reported to bind to the nematode cuticle or to host glycans, or expression was elevated during parasitism. These observations are an indication of a possible role in interfering with recognition or host defenses [17,69,70]. Also, galectin-like proteins were found in the secretions of several parasitic nematodes [71,72,73]. In addition, some of these galectin-like proteins were found to inhibit induced inflammation reactions in host organisms and are able to bind host immune cells [74,75].

Calreticulins have been reported in both plant and animal parasitic nematodes, where they are able to modulate the defense responses of the host [66,76,77]. Besides that, calreticulin is also involved in reproduction and abiotic stress responses [78,79]. To our knowledge, C-type lectins, galectins, and calreticulins are the only types of lectins that have been studied in nematodes so far. We have shown that these types of lectins are present in all investigated nematode species, but that there are also other interesting lectins awaiting further characterization.

The large variety of lectin-like proteins in nematodes may indicate their involvement in different processes, varying from immune responses to cellular growth and cell–cell interaction [1]. We have included lectins related to class V chitinase homologues (CRA) in our search. This type of lectin has a high sequence and structural similarity to true class V chitinases. In addition, although no chitinase activity is present in CRA lectins, the catalytic motif might be preserved [80]. Only experimental evidence showing carbohydrate-binding activity and lack of chitinase activity can give a decisive answer regarding the true nature of the protein: lectin or chitinase?

Potential M-type lectins were subjected to a thorough analysis before identifying them as lectins. M-type lectins are members of the glycoside hydrolase family 47 and are highly similar to α-mannosidases. Although M-type lectins can bind mannose glycans, they lack the catalytic residues and disulfide bond needed to trim the glycans. M-type lectins work in association with mannosidases and lectins from the calnexin group to supervise the correct folding of newly synthesized glycoproteins in the endoplasmic reticulum [81,82]. This process has been investigated in mammals, but key components of the machinery are present in *C. elegans* as well [83]. Mammals are known to have three M-type lectins, annotated as EDEM1-3 (ER degradation enhancing α-mannosidase-like protein) [84]. *C. elegans* also has three M-type lectins as homologues for EDEM1-3, but it seems that the investigated parasitic nematode species can contain up to six EDEM homologues. The discrepancy in the number of EDEM homologues compared to mammals and *C. elegans* seems not to be that rare, since budding yeast and *Drosophila* have one and two EDEM homologues, respectively [85,86].

A curious observation was the discovery of proteins with a hevein-like domain in *C. elegans*. This small chitin-binding monomeric protein is involved in the coagulation of latex and has antifungal properties [49,87]. Hevein-like carbohydrate-binding domains seem to be omnipresent in the plant kingdom and have been reported in fungi as well [6]. Although animal proteins with a similar three-dimensional structure have been described, this is the first time hevein-like peptides have been reported in animals [88,89]. Nematode hevein-like domains contain eight conserved cysteine residues. Two out of three aromatic amino acid residues involved in chitin binding are conserved in nematode sequences; only the first aromatic residue is replaced by alanine or glycine. It should be noted that the second conserved aromatic residue is changed from tryptophan to tyrosine in nematodes. NMR investigations have shown that a Tryptophan/Tyrosine substitution does not necessarily affect the carbohydrate-binding properties of the hevein-like domain [90], but experimental studies are needed to investigate the chitin-binding abilities. The antifungal properties of the original hevein peptide were described in 1991 [87]. Since then, several hevein-like peptides have been isolated from other plant species and their antifungal properties have been confirmed [91]. Also, oomycete growth can be inhibited by hevein-like peptides [92]. Since most oomycetes do not contain chitin in their cell wall, the antifungal activity may not be solely based upon chitin binding. This was confirmed for hevein-like peptides from wheat, which were shown to have proteinase inhibitor properties, thereby inhibiting fungal growth [93]. The function of hevein-like domain-containing proteins remains to be elucidated. It is possible that nematodes use these proteins as some sort of protection against harmful fungi or bacteria. This hypothesis is supported by the fact that two hevein-like domain-containing genes from *C. elegans* (T01C4.1 and T13H5.3) were found to be differentially regulated upon bacterial or fungal infection [94].

A reciprocal blast of all retrieved CTLDs revealed that three domains are present in all seven investigated nematode genomes. This implies that these domains are not related to a specific lifestyle of the nematode, but rather that they have a more general function. Previous studies have shown that all three domains are differentially regulated upon bacterial or fungal infection of *C. elegans*. F32E10.3 is upregulated after infection with *Photorhabdus luminescens* and *Serratia marcescens* [94,95], but the abundance of the protein is lower after infection with *Bacillus thuringiensis* [96]. F47C12.2 is upregulated by infection with *P. luminescens* and *Enterococcus faecalis*, but downregulated by *S. marcescens* and *Drechmeria coniospora* [94]. The expression of F25B4.9, on the other hand, was downregulated upon infection with four different pathogens: *S. marcescens*, *D. coniospora*, *Xenorhabdus nematophila*, and *Pseudomonas aeruginosa* [97,98,99]. In addition, the corresponding protein product was less abundant upon *B. thuringiensis* infection [96]. All these results point towards a link with the innate immune system for these three common C-type lectins.

The C-type lectin family seems to be the most abundant lectin family in all types of nematodes. The C-type lectin family in *C. elegans* and other free-living nematodes is much larger compared to nematodes with a parasitic lifestyle. The number of 252 predicted members of this family is much higher than the 125 initially predicted family members [18], but it resembles the number of CTLD coding genes described by Schulenburg et al. [19] and Pees et al. [20]. While Drickamer et al. [18] identified CTLDs in *C. elegans* using a general profile, Schulenburg et al. [19] constructed a *C. elegans* specific profile, hence identifying more potential CTLD containing genes. In this research a general nematode profile was predicted, causing a small difference in number of CTLDs retrieved from the *C. elegans* genome. Taxon-specific profiles can be designed to get a more precise picture of lectin-like proteins in separate nematode species. Looking at CTLDs with predicted carbohydrate-binding properties, the ratio of *C. elegans* sequences compared to non-free-living nematodes was not altered. Genome sizes could not explain the difference in CTLD content between the different nematodes. Table S1 also shows genome sizes for each nematode. A Pearson’s correlation test indicated that there is no correlation between genome size and number of CTLDs present.

It was noticed that all free-living nematodes possess more CTLD-containing genes compared to nematode species with a different lifestyle. The only exception is the free-living nematode *Rhabditophanes*. It should be noted here that *Rhabditophanes* is classified as a free-living nematode, but it is placed within a clade of animal parasitic nematodes. This suggests that it has reverted to a non-parasitic lifestyle, which may explain the difference in CTLD content [100]. Besides the free-living species, some entomopathogenic species (*Steinernema*) also appear to have a large number of CTLD-containing genes in their genome. Curiously, only 15 CTLD-containing genes were found in the genome of the entomopathogenic nematode *Heterorhabditis bacteriophora*. Both free-living and insect-parasitic nematodes spend a large share of their lifecycle in soil or decaying organic matter, so they are prone to infections from the vast bacterial and fungal communities in those habitats. The large number of CTLD-containing proteins might help in giving the innate immune system an extra boost as protection against a harmful environment. Animal and plant parasitic nematodes are partially protected by the host environment, thus may need fewer CTLD-containing proteins supporting an adequate immune system. The important role of some of these proteins in the innate immune systems has already been described in *C. elegans* [19]. It should be noted that this hypothesis is based upon differential expression of CTLD-containing genes upon bacterial infection. Since experimental validation is lacking, the exact function in innate immunity remains unclear. This issue is raised and described by Pees et al. [20]. While *Steinernema* spp. and *Heterorhabditis* spp. occupy the same habitat and share similarities in morphology, lifecycle, and bacterial symbiosis, there is a large difference in the number of CTLD-containing genes. Although there are some contradicting studies, these similarities can probably be attributed to convergent evolution (reviewed by [101]). This hypothesis is supported by more recent phylogenetic analyses putting the two genera in different clades [51]. Convergent evolution might be an explanation for the discrepancy in CTLD content in these two genera. The genome of only one *Heterorhabditis* species was investigated. Results should be confirmed with the genome data of additional *Heterorhabditis* species (e.g., *Heterorhabditis megidis*, *Heterorhabditis indica*, and *Heterorhabditis sonorensis*) which might be available in the near future.

This research showed that several lectin folds are present over different nematode species. Although these typical lectin folds point towards carbohydrate-binding properties, this assumption cannot be made without any further investigations. Some lectin-like domains do not bind sugars or are known to bind other ligands instead. For instance, protein domains with a C-type lectin-like fold appear to be protein binding [102], while others are shown to bind lipids [103] or might bind inorganic compounds [104]. This encouraged us to subject the retrieved lectin-like domains to a more detailed investigation. Protein sequences were examined for the presence of certain key amino acid residues involved in carbohydrate binding in each type of lectin domain. Although this analysis gives an indication about the carbohydrate-binding properties of the respective lectin-like domains, experimental evidence is needed as confirmation. The domain search of potential carbohydrate-binding lectins indicated that most potential carbohydrate-binding lectin domains occur as sole domain or as tandem repeat in a protein. The fact that the remaining part of potential carbohydrate-binding domains is combined with a variety of other protein domains is in accordance with the observation that lectin-like proteins are involved in a multitude of processes [3].

This research has provided new information regarding the distribution and conservation of lectin domain-containing genes in the phylum Nematoda. Next to animal lectins, a typical plant lectin domain was also retrieved from the genome of some nematode species: the hevein domain. Functional characterization is needed to elucidate the role of the hevein domain in this phylum. Results have also confirmed that the C-type lectin family is the most abundant lectin family in nematodes and that it is much larger in free-living and some entomopathogenic nematodes. Further research is needed to characterize these C-type lectins regarding their function and carbohydrate-binding properties.

## 4. Materials and Methods

### 4.1. Lectin-Like Domain Discovery

Available Pfam profiles were downloaded for 11 plant lectins: *Agaricus bisporus* agglutinin homologs (PF07367), Amaranthins (PF07468), Class V chitinases (PF00704), Cyanovirin (PF08881), *Galanthus nivalis* agglutinin (PF01453), Hevein (PF00187), Jacalins (PF01419), Legume-like lectin (PF00139), LysM (PF01476), Nictaba (PF14299), and Ricin-B (PF00652). Six Pfam profiles of animal lectins were downloaded as well: Calnexin/calreticulin (PF00262), M-type lectins (PF01532), P-type lectins (PF02157), C-type lectins (PF00059), galectins (PF00337), and I-type lectins (PF07686). Two additional animal lectins share the same Pfam profile with plant lectins: R-type lectins (PF00652) and L-type lectins (PF00139). Intelectins and F-type lectins are two animal-type lectins for which no Pfam profile is available. Characterized sequences per lectin type were downloaded from www.imperial.ac.uk/research/animallectins, serving as seeds to design a new hmm profile for both lectin types. Profiles were built with the Hmmbuild function of HMMER v3.0 (http://hmmer.org) using a Stockholm alignment of the seed sequences as input. Data were scanned with profiles of bacterial lectins as well: LecA (PF07828), LecB (PF07472), FimH (PF09160), PapG (PF03627), F17G (PF09222), Verotoxin (PF02258), Hemolysin (PF16458), and Intimin (PF07979).

Hmmsearch (HMMER v3.0) was used to query predicted protein sequences from nematode genome databases with the 27 profiles (bitscore > 15). Sequence data were used from *Caenorhabditis elegans*, [63], *Brugia malayi* [105], *Ascaris suum* [106], *Globodera pallida* [107], *Bursaphelenchus xylophilus* [108], *Meloidogyne hapla* [109], and *Meloidogyne incognita* [110]. All hits were blasted against the Swissprot database (blastp) to help with the selection of true hits. All hits were checked manually to reduce the number of false positives. A phylogenetic tree of the M-type lectin domains was constructed with the maximum likelihood method using BioEdit (Ibis Biosciences, Carlsbad, CA, USA) [111]. Potential transmembrane regions were predicted with the TMHMM Server v. 2.0 [112].

Lectin-like domains in nematode hits were extracted and aligned with ClustalO [113] to construct a new HMM profile for each type of lectin with HMMER. These new profiles were used to query the different nematode genome databases again (bitscore > 20). In addition, transcriptome databases of some plant parasitic nematodes were searched for lectin-like domains as well: *Hirschmanniella oryzae* [114], *Meloidogyne graminicola* [115], *Heterodera glycines* (http://nematode.net), *Heterodera avenae* [116], *Pratylenchus coffeae* [117], and *Pratylenchus thornei* [118]. All new hits were checked separately to reduce the number of false positives. The nematode-specific profile was used on additional nematode genomes downloaded from Wormbase Parasite [119].

### 4.2. Structural Classification

Nematode proteins predicted to have a CTLD were used in a HMMer search against the Pfam database to predict protein domains. The presence of the different domains was used to make a structural classification based upon the organization of Drickamer et al. [18].

### 4.3. Conserved Sequences

A reciprocal blast (blastp, bitscore 50) was performed to search for protein sequences containing a CTLD that is conserved among several species. The reciprocal blast was carried out using the full protein sequences.

### 4.4. Phylogenetic Analysis

Potential carbohydrate-binding CTLDs were aligned with ClustalO [113] Model selection was done in TOPALi v2.5 [120]. According to the BIC criterion, a WAG substitution model of protein evolution was used. A phylogenetic tree was estimated using the RAxML webserver program [121]. A maximum likelihood search was performed with a total of 100 bootstrap replications. The predicted tree was visualized with Figtree (University of Edinburgh, Edinburgh, UK).

## 5. Conclusions

A thorough research of several nematode genomes provides us with a clearer image of the presence of several classes of potential lectins. Results from this analysis showed that C-type lectin-like proteins as well as galectin-like proteins are more prevalent compared to other lectin-like proteins in nematodes. Free living nematodes possess more C-type lectin-like proteins compared to nematodes with an alternative life style. Not only the typical animal lectin classes are present, but also some plant-type lectins, one of them being hevein. So far, hevein-type lectins have not been reported in animals, which makes it an interesting topic for further investigation. Although we have provided a list with possible lectin proteins in nematodes, their potential carbohydrate-binding properties still await further investigation and experimental confirmation.

## Figures and Tables

**Table 1:** Results of the profile hidden Markov model (HMMer) search. Numbers indicate the number of sequences with one or more domains of a type of lectin domain in each nematode species. The total number of sequences per lectin domain is indicated in the bottom row. Numbers in brackets give an indication of the number of potential carbohydrate-binding lectins, a question mark (?) indicates that we are unable to draw conclusions regarding carbohydrate binding properties. CE: *Caenorhabditis elegans*, AS: *Ascaris suum*, BM: *Brugia malayi*, BX: *Bursaphelenchus xylophilus*, GP: *Globodera pallida*, HA: *Heterodera avenae*, MI: *Meloidogyne incognita*, MH: *Meloidogyne hapla*, MG: *Meloidogyne graminicola*, HO: *Hirschmanniella oryzae*, HG: *Heterodera glycines*, PC: *Pratylenchus coffeae*, PT: *Pratylenchus thornei*, #Seq: the number of protein sequences that was scanned for potential lectin domains. # indicates the total number of potential lectins for each class.

Species		#Seq	Class V Chitinase	Hevein	Legume	LysM	F-Type Lectin	Ricin-B	Calnexin/Calreticulin	M-Type Lectin	C-Type Lectin	Galectin
Genomes	CE	26150	34 (30)	3	2 (2)	8 (?)	1 (0)	14 (6)	2 (2)	3	252 (52)	38 (18)
	AS	18542	8 (4)	0	3 (2)	2 (?)	1 (0)	9 (8)	3 (2)	5	36 (8)	11 (6)
	BM	17938	12 (7)	0	2 (2)	4 (?)	0	11 (10)	8 (5)	4	18 (1)	13 (10)
	BX	18074	7 (1)	0	2 (2)	1 (?)	0	11 (7)	3 (1)	4	15 (0)	14 (4)
	GP	16417	9 (6)	0	3 (2)	0	0	9 (6)	3 (2)	5	22 (4)	38 (7)
	MI	20359	2 (0)	0	1 (1)	3 (?)	0	7 (5)	4 (3)	6	57 (8)	70 (9)
	MH	14421	4 (3)	0	2 (2)	4 (?)	0	8 (5)	1 (0)	4	29 (2)	53 (3)
Transcriptomes	MG	10973	3	0	0	0	0	1	4	2	48	13
	HO	17061	5	0	2	2	0	2	5	2	33	28
	HA	10070	3	0	2	2	0	6	1	4	1	9
	HG	12262	4	0	0	1	0	0	5	0	4	9
	PC	11507	0	0	0	1	0	2	1	0	3	7
	PT	19533	0	0	0	1	0	4	2	1	0	5
	#		91	3	19	29	2	84	42	40	518	308

**Table 2:** Organization of proteins from the investigated nematode species containing CTLDs. An asterisk (*) indicates a group that was not previously characterized [20]. CTLD: C-type lectin-like domain, CE: *C. elegans*, AS: *A. suum*, BM: *B. malayi*, GP: *G. pallida*, MI: *M. incognita*, MH: *M. hapla*, BX: *B. xylophilus*. CUB: Complement C1r/C1s, Uegf, Bmp1 domain, CW: conserved cysteine and trypthophan residues, also known as PAN-3 domain, VWA: Von Willebrand factor type A domain. Complex structure: CTLD-containing gene with multiple domains where CTLD might not determine the actual function of the protein.

Class	Description	CE	AS	BM	GP	MI	MH	BX
I	1 CTLD	141	24	15	17	27	17	9
II	2–3 CTLD	46	0	0	1	24	10	1
III	1–3 CTLD + 1–3 CUB	28	0	1	0	0	1	1
IV	1–2 CTLD + 1–2 CW	16	0	0	0	0	0	0
V	1 CTLD + 1–2 VWA	14	9	0	0	0	0	0
VI	Complex structure	7	2	1	4	6	1	4
VII*	4 CTLD	0	1	0	0	0	0	0

**Table 3:** Presence of conserved residues in nematode CTLDs. The first column shows the nine different conserved residues. The second column indicates the share of sequences that contain the respective residue. The last three rows show the share of the different combinations of disulfide bridges in the investigated nematode sequences. C1-C4: conserved cysteine residues forming the disulfide bridges (C1-C4 and C2-C3 bridge). W, G, and L: conserved residues from the “WIGL” motif. α1A: conserved alanine residue in the first α-helix of the C-type lectin fold. β1’L: conserved leucine residue in the first β-sheet of the C-type lectin fold.

Conserved Residue	% Residue
C1	93.17
C2	68.45
C3	70.30
C4	77.49
W	70.66
G	90.59
L	42.80
α1A	80.07
β1’L	64.94
Both C bridges	48.71
Only C1-C4 bridge	24.35
Only C2-C3 bridge	11.99

## Supplementary Materials

Supplementary materials can be found at www.mdpi.com/1422-0067/18/1/91/s1.

## Abbreviations

CTLD	C-type lectin-like domain
CE	*C. elegans*
BM	*B. malayi*
AS	*A. suum*
MI	*M. incognita*
MH	*M. hapla*
MG	*M. graminicola*
BX	*B. xylophilus*
GP	*G. pallida*
HA	*H. avenae*
HG	*H. glycines*
HO	*H. oryzae*
PC	*P. coffeae*
PT	*P. thornei*
Trp	Tryptophan
Tyr	Tyrosine
GHF	Glycosyl hydrolase family
HMM	Hidden Markov model
EDEM	ER degradation-enhancing α-mannosidase-like protein
