# Supplementary material for: The Distribution of Lectins across the Phylum Nematoda: A Genome-Wide Search
Source: Int J Mol Sci. 2017 Jan 4;18(1):91. doi: 10.3390/ijms18010091 (PMC5297725; doi:10.3390/ijms18010091)
Supplement: Supplementary file 1 [file ijms-18-00091-s001.pdf]

Supplementary Materials: The Distribution of Lectins across the Phylum Nematoda: A Genome-Wide Search

Lander Bauters, Diana Naalden and Godelieve Gheysen

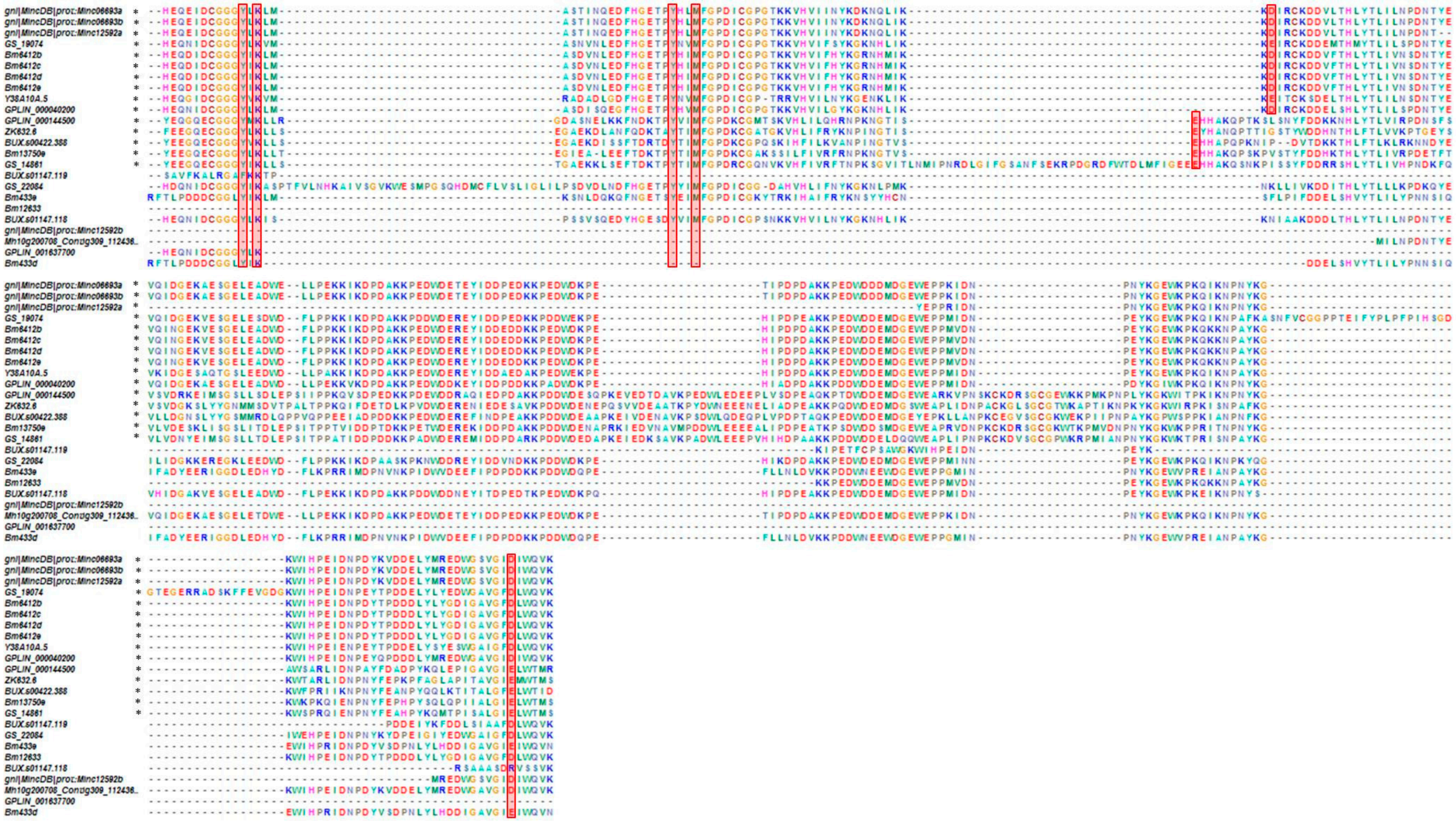

Figure S1. Alignment of partial calreticulin/calnexin sequences. Amino acids are represented by one letter codes in different colors. Residues needed for carbohydrate binding are indicated in red boxes. Sequences containing all six necessary residues are indicated with an asterisk.

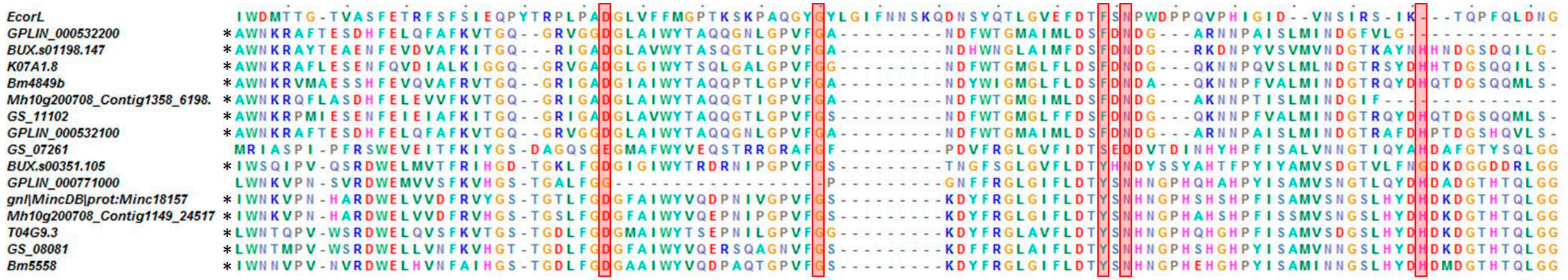

Figure S2. Alignment of partial legume lectin-like sequences. Amino acids are represented by one letter codes in different colors. EcorL is a legume lectin originating from *Erythrina corallodenron*, used in this alignment to compare carbohydrate binding sites. The residues necessary for carbohydrate interaction are shown in red boxes. Nematode lectin-like sequences containing at least four out of five key residues are indicated with an asterisk.

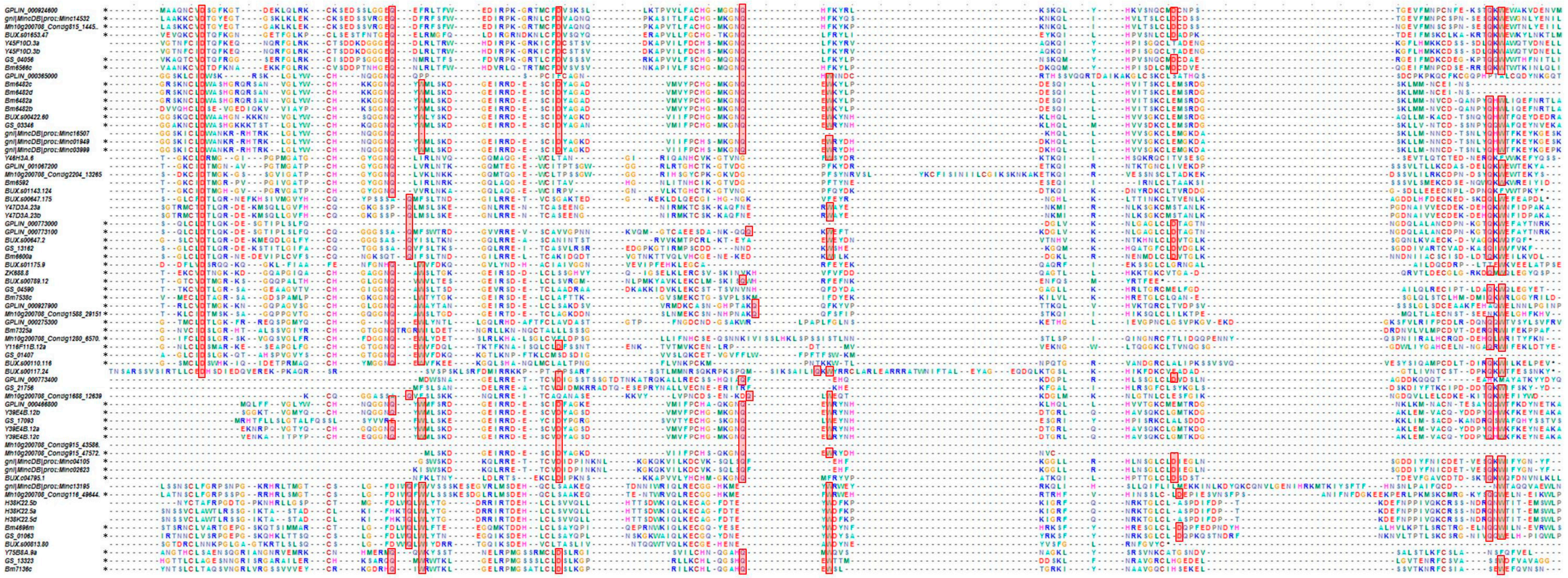

Figure S3. Alignment of possible Ricin-B lectin-like domains. Amino acids are represented by one letter codes in different colors. The key amino acid residues (D-Q-W) involved in carbohydrate binding, which are repeated three times, are boxed in red. Sequences that have at least one complete D-Q-W triad are indicated with an asterisk.

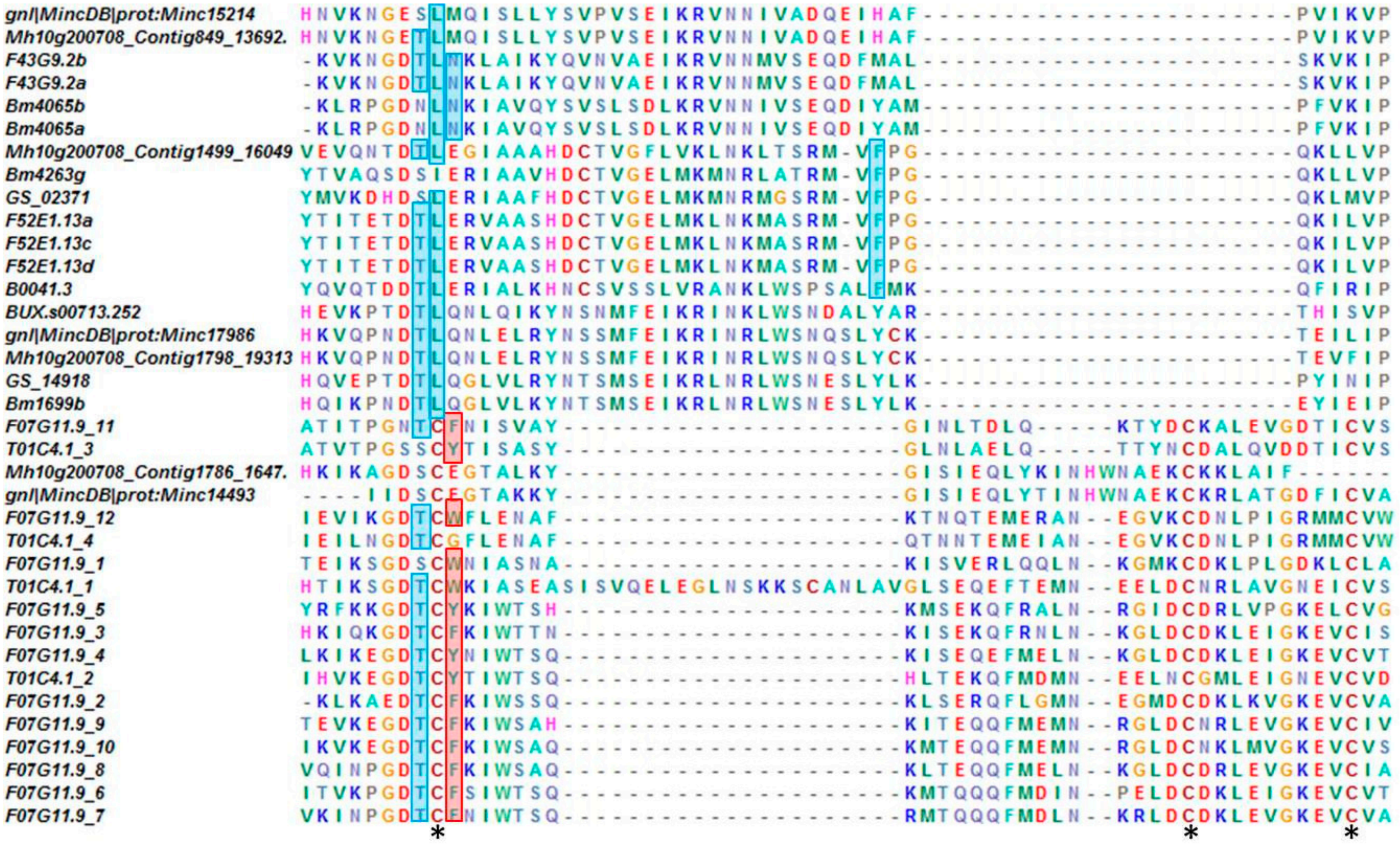

Figure S4. Alignment of possible LysM lectins. Amino acids are represented by one letter codes in different colors. Conserved cysteine residues are marked with an asterisk under the alignment. The key residue involved in carbohydrate binding in an eukaryote is boxed in red [1]. The conserved key residues characterized in bacterial sequences are boxed in blue [2].

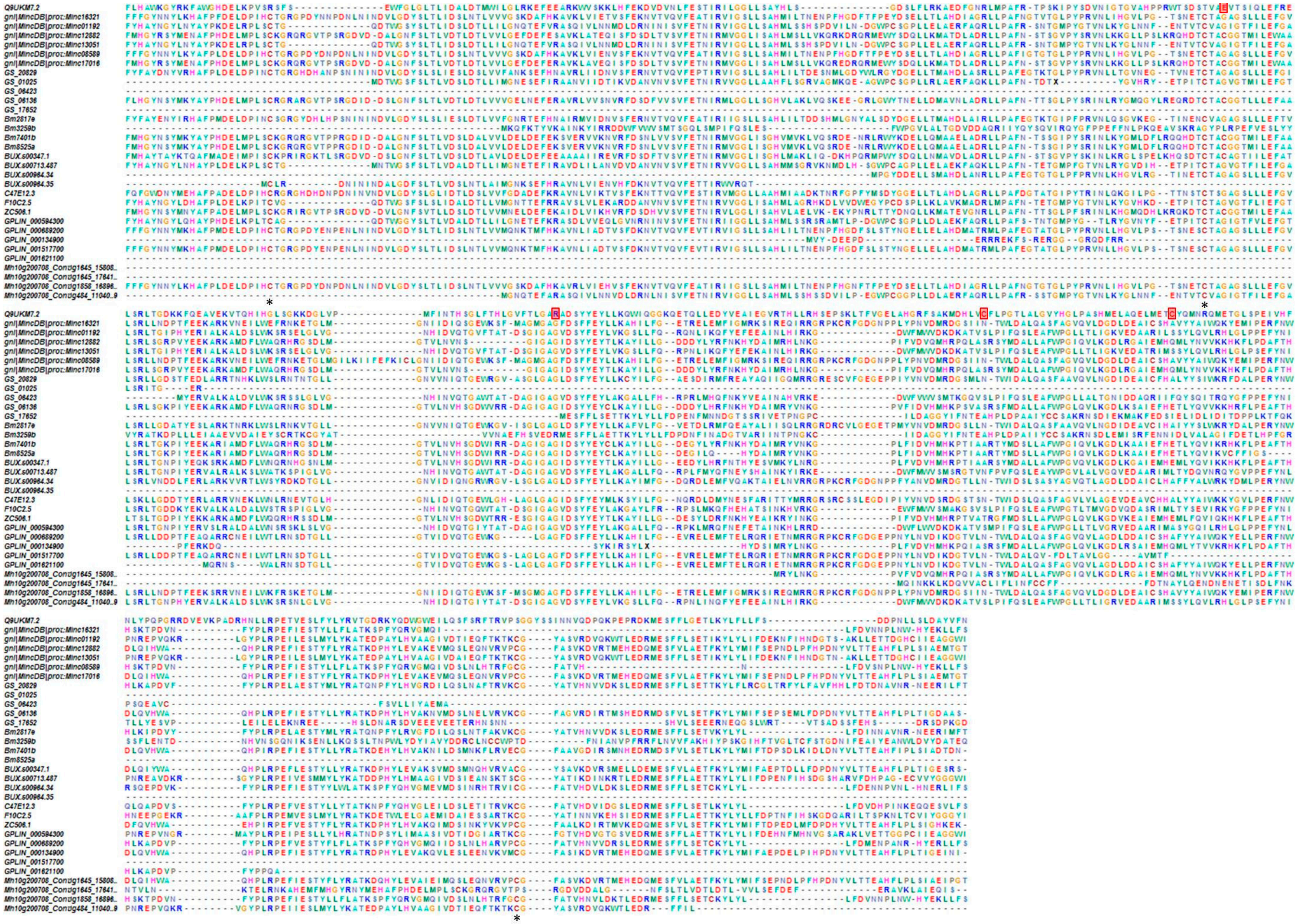

Figure S5. Alignment of potential M-type lectins. Amino acids are represented by one letter codes in different colors. M-type lectin-like sequences are compared with a human alpha-mannosidase (Q9UKM7.2). Conserved cysteine residues in M-type lectin-like sequences are denoted with an asterisk under the alignment. Key residues necessary for mannosidase activity are boxed in red. These residues are absent in M-type lectins.

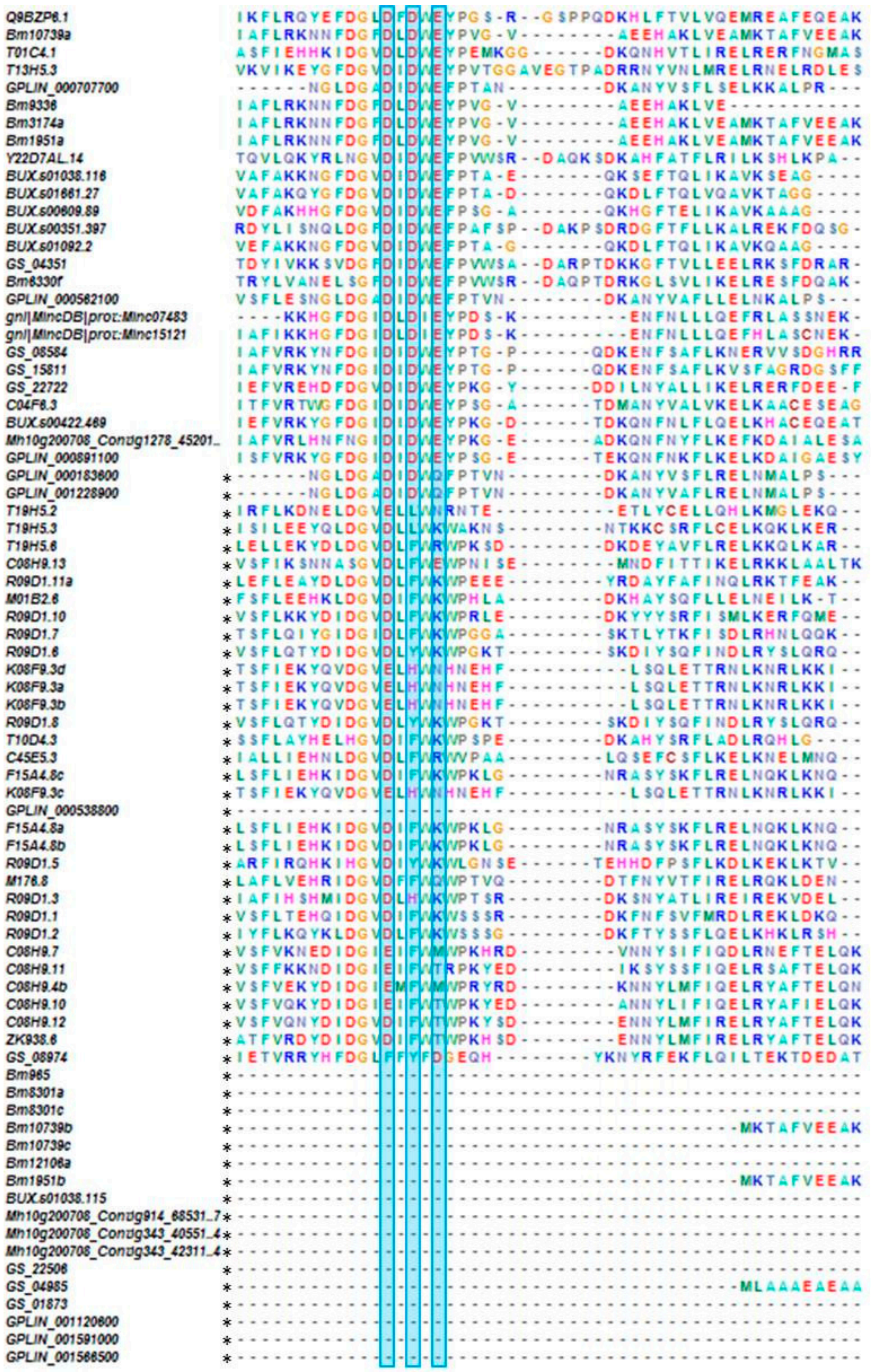

Figure S6. Alignment of the catalytic site of GHF18 chitinases. Amino acids are represented by one letter codes in different colors. The three catalytic residues (DXDXE) are boxed in blue. Sequences that do not contain all three key residues are marked with an asterisk. Marked sequences may be considered as potential chitinase-like lectins. The top sequence is a reference human GHF18 chitinase (Q9BZP6.1).

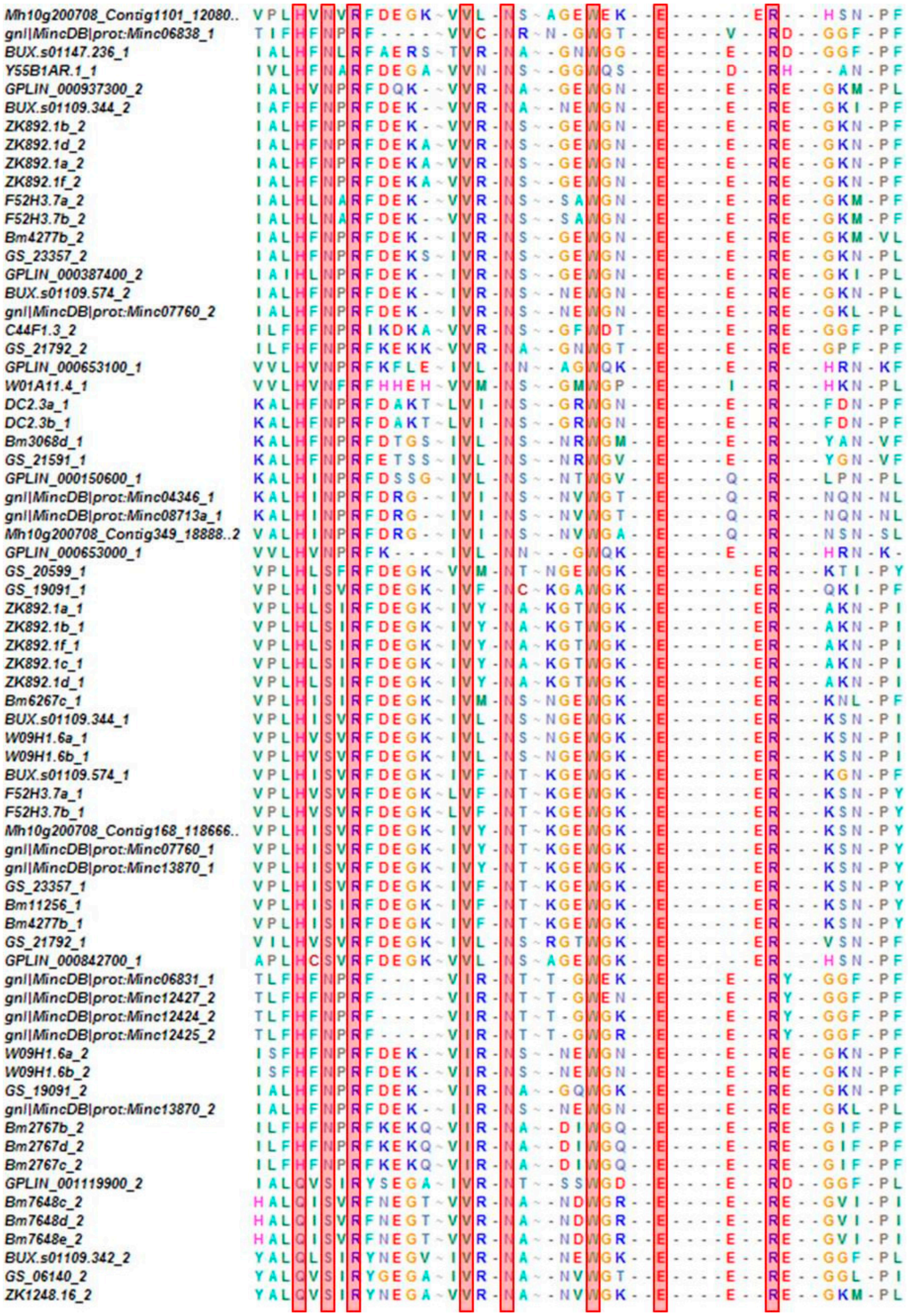

Figure S7. Alignment of the carbohydrate binding region of potential galectin sequences. Amino acids are represented by one letter codes in different colors. Only sequences containing all eight essential residues for carbohydrate binding are shown here. Key residues are boxed in red.

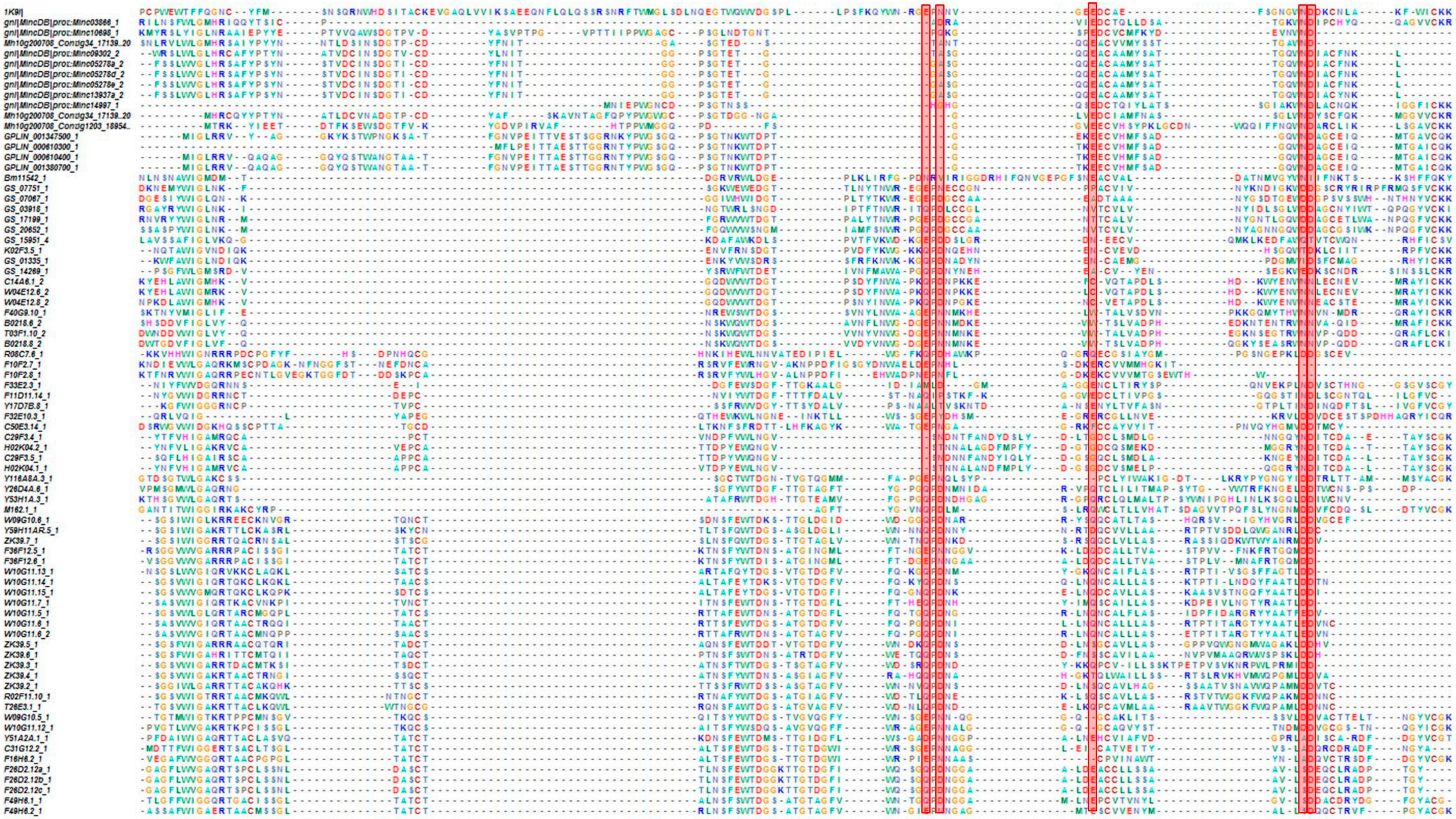

Figure S8. Alignment of 77 potential carbohydrate-binding C-type lectin-like sequences. Amino acids are represented by one letter codes in different colors. Sequences were compared with a bacterial C-type lectin domain (1K9I). Key residues involved in carbohydrate binding are boxed in red. CTLDs were considered as potentially carbohydrate binding if at least three out of the five key residues were conserved.

Table S1. Presence of genes containing C-type lectin domains or hevein-like domains in different nematode genomes. This table gives an overview for several nematodes of which the genome is known and indicates the number of C-type lectin domain and hevein-like domain-containing genes within each genome. Data were downloaded from the Wormbase-Parasite website [3]. Nematode genomes used in the main analysis are in gray.

Species	Genome Size (Mb)	Life Style	Number of C-Type Lectin Domain Containing Genes	Number of Hevein-Like Domain Containing Genes	Phylogenetic Cluster (According to [4])	Provider	Reference
Acanthocheilonema viteae	77	Animal parasitic	14	0	8b	Blaxter laboratory at University of Edinburgh	
Ancylostoma ceylanicym	313	Animal parasitic	72	2	9b	Cornell University	[5]
Ancylostoma duodenale	333	Animal parasitic	76	3	9b	Mitreva laboratory at the Genome Institute of Washington University	
Ancylostoma caninum	466	Animal parasitic	81	2	9b	Mitreva laboratory at the Genome Institute of Washington University	[6]
Angiostrongylus costaricensis	263	Animal parasitic	8	0	9c	Parasite Genomic group at the Wellcome Trust Sanger Institute	
Angiostrongylus cantonensis	253	Animal parasitic	7	0	9c	Parasite Genomic group at the Wellcome Trust Sanger Institute	
Anisakis simplex	126	Animal parasitic	34	0	8b	Parasite Genomic group at the Wellcome Trust Sanger Institute	
Ascaris lumbricoides	317	Animal parasitic	29	0	8b	Parasite Genomic group at the Wellcome Trust Sanger Institute	
Brugia pahangi	91	Animal parasitic	13	0	8b	Parasite Genomic group at the Wellcome Trust Sanger Institute	
Brugia timori	65	Animal parasitic	10	0	8b	Parasite Genomic group at the Wellcome Trust Sanger Institute	
Caenorhabditis sinica	132	Free living	399	6	9a	Blaxter laboratory at University of Edinburgh	[7]
Caenorhabditis japonica	166	Free living	104	6	9a	Washington University	[7]
Caenorhabditis brenneri	190	Free living	264	5	9a	Washington University	[7]
Caenorhabditis tropicalis	79	Free living	221	5	9a	Washington University	[7]
Caenorhabditis angaria	106	Free living	261	3	9a	California Institute of Technology	[8]
Caenorhabditis briggsae	108	Free living	160	3	9a	Sanger Institute	[9]
Caenorhabditis remanei	145	Free living	235	2	9a	Washington University	[7]
Cylicostephanus goldi	173	Animal parasitic	30	0	x	Parasite Genomic group at the Wellcome Trust Sanger Institute	
Dictyocaulus viviparus	169	Animal parasitic	7	1	9B	Blaxter laboratory at University of Edinburgh	[10]
Dirofilaria immitis	88	Animal parasitic	20	0	8b	Blaxter laboratory at University of Edinburgh	[11]
Dracunculus medinensis	104	Animal parasitic	23	0	8b	Parasite Genomic group at the Wellcome Trust Sanger Institute	
Elaeophora elaphi	83	Animal parasitic	13	0	8b	Parasite Genomic group at the Wellcome Trust Sanger Institute	

Table S1. *Cont.*

Species	Genome Size (Mb)	Life Style	Number of C-Type Lectin Domain Containing Genes	Number of Hevein-Like Domain Containing Genes	Phylogenetic Cluster (According to [4])	Provider	Reference
Enterobius vermicularis	150	Animal parasitic	80	1	8a	Parasite Genomic group at the Wellcome Trust Sanger Institute	
Gongylonema pulchrum	322	Animal parasitic	24	0	8b	Parasite Genomic group at the Wellcome Trust Sanger Institute	
Haemonchus contortus	370	Animal parasitic	87	1	9b	Parasite Genomic group at the Wellcome Trust Sanger Institute	[12]
Haemonchus placei	259	Animal parasitic	34	1	9b	Parasite Genomic group at the Wellcome Trust Sanger Institute	
Heligmosomoides polygyrus	561	Animal parasitic	36	0	9b	Parasite Genomic group at the Wellcome Trust Sanger Institute	
Heterorhabditis bacteriophora	77	Insect parasitic	15	1	9b	Mitreva laboratory at the Genome Institute of Washington University	[13]
Litomosoides sigmodontis	65	Animal parasitic	17	0	8b	Blaxter laboratory at University of Edinburgh	
Loa loa	96	Animal parasitic	15	0	8b	Institute for Genome Sciences at the University of Maryland	[14]
Necator americanus	244	Animal parasitic	49	1	9b	Mitreva laboratory at the Genome Institute of Washington University	[15]
Nippostrongylus brasiliensis	294	Animal parasitic	30	1	9b	Parasite Genomic group at the Wellcome Trust Sanger Institute	
Oesophagostomum dentatum	490	Animal parasitic	76	1	9b	Mitreva laboratory at the Genome Institute of Washington University	
Onchocerca volvulus	96	Animal parasitic	17	0	8b	Parasite Genomic group at the Wellcome Trust Sanger Institute	[7]
Onchocerca ochengi	112	Animal parasitic	13	0	8b	Parasite Genomic group at the Wellcome Trust Sanger Institute	
Onchocerca flexuosa	86	Animal parasitic	10	0	8b	Parasite Genomic group at the Wellcome Trust Sanger Institute	
Panagrellus redivivus	65	Free living	134	1	10b	California Institute of Technology	[16]
Parascaris equorum	185	Animal parasitic	10	0	8b	Parasite Genomic group at the Wellcome Trust Sanger Institute	
Parastrongyloides trichosuri	42	Animal parasitic	29	1	10b	Parasite Genomic group at the Wellcome Trust Sanger Institute	
Pristionchus pacificus	172	Free living	151	2	9a	Max-Planck Institute for Developmental Biology	[17]
Pristionchus exspectatus	177	Free living	116	1	9a	Max-Planck Institute for Developmental Biology	
Rhabditophanes kr3021	47	Free living	34	1	10b	Parasite Genomic group at the Wellcome Trust Sanger Institute	
Romanomermis culicivorax	323	Insect parasitic	25	0	2a	University of Cologne	[18]
Soboliphyme baturini	218	Animal parasitic	4	0	2a	Parasite Genomic group at the Wellcome Trust Sanger Institute	
Steinernema scapterisci	80	Insect parasitic	295	2	10a	California Institute of Technology	[19]
Steinernema carpocapsae	86	Insect parasitic	187	2	10a	California Institute of Technology	[19]

Table S1. *Cont.*

Species	Genome Size (Mb)	Life Style	Number of C-Type Lectin Domain Containing Genes	Number of Hevein-Like Domain Containing Genes	Phylogenetic Cluster (According to [4])	Provider	Reference
Steinernema feltiae	83	Insect parasitic	241	1	10a	California Institute of Technology	[19]
Steinernema monticolum	89	Insect parasitic	291	0	10a	California Institute of Technology	[19]
Steinernema glaseri	93	Insect parasitic	162	0	10a	California Institute of Technology	[19]
Strongyloides venezuelensis	52	Animal parasitic	28	1	10b	Parasite Genomic group at the Wellcome Trust Sanger Institute	[20]
Strongyloides ratti	43	Animal parasitic	27	1	10b	Parasite Genomic group at the Wellcome Trust Sanger Institute	[20]
Strongyloides stercoralis	43	Animal parasitic	27	1	10b	Parasite Genomic group at the Wellcome Trust Sanger Institute	[20]
Strongyloides papillosus	60	Animal parasitic	31	0	10b	Parasite Genomic group at the Wellcome Trust Sanger Institute	[20]
Strongylus vulgaris	291	Animal parasitic	32	1	9b	Parasite Genomic group at the Wellcome Trust Sanger Institute	
Syphacia muris	99	Animal parasitic	52	1	8a	Parasite Genomic group at the Wellcome Trust Sanger Institute	
Teladorsagia circumcincta	700	Animal parasitic	93	2	9b	Mitreva laboratory at the Genome Institute of Washington University	
Thelazia callipaeda	75	Animal parasitic	11	0	8b	Parasite Genomic group at the Wellcome Trust Sanger Institute	
Toxocara canis	300	Animal parasitic	27	0	8b	Parasite Genomic group at the Wellcome Trust Sanger Institute	
Trichinella spiralis	66	Animal parasitic	9	0	2a	Mitreva laboratory at the Genome Institute of Washington University	[21]
Trichinella nativa	49	Animal parasitic	7	0	2a	Mitreva laboratory at the Genome Institute of Washington University	
Trichuris muris	84	Animal parasitic	6	0	2a	Parasite Genomic group at the Wellcome Trust Sanger Institute	[22]
Trichuris suis	64	Animal parasitic	5	0	2a	Mitreva laboratory at the Genome Institute of Washington University	
Wuchereria bancrofti	77	Animal parasitic	17	0	8b	Parasite Genomic group at the Wellcome Trust Sanger Institute	
Caenorhabditis elegans	100	Free living	252	3	9a		[23]
Brugia malayi	88	Animal parasitic	18	0	8b	TIGR Institute	[24]
Ascaris suum	266	Animal parasitic	36	0	8b	Davis laboratory at the University of Colorado	[25]
Globodera pallida	124	Plant parasitic	22	0	12b	Parasite Genomic group at the Wellcome Trust Sanger Institute	[26]
Meloidogyne hapla	53	Plant parasitic	29	0	12b	Plant Nematode Genomics group at North Carolina State University	[27]
Meloidogyne incognita	86	Plant parasitic	57	0	12b	French National Institute for Agricultural Research (INRA)	[28]
Bursaphelenchus xylophilus	75	Plant parasitic	15	0	10b	Parasite Genomic group at the Wellcome Trust Sanger Institute	[29]

References

1. Gerken, T.A.; Revoredo, L.; Thome, J.J.; Tabak, L.A.; Vester-Christensen, M.B.; Clausen, H.; Gahlay, G.K.; Jarvis, D.L.; Johnson, R.W.; Moniz, H.A.; et al. The lectin domain of the polypeptide GalNAc transferase family of glycosyltransferases (ppGalNAc Ts) acts as a switch directing glycopeptide substrate glycosylation in an N- or C-terminal direction, further controlling mucin type *O*-glycosylation. *J. Biol. Chem.* **2013**, *288*, 19900–19914.
2. Ohnuma, T.; Onaga, S.; Murata, K.; Taira, T.; Katoh, E. LysM domains from *Pteris ryukyuensis* chitinase-A: A stability study and characterization of the chitin-binding site. *J. Biol. Chem.* **2008**, *283*, 5178–5187.
3. Nicol, P.; Gill, R.; Fosu-Nyarko, J.; Jones, M.G. De novo analysis and functional classification of the transcriptome of the root lesion nematode, *Pratylenchus thornei*, after 454 GS FLX sequencing. *Int. J. Parasitol.* **2012**, *42*, 225–237.
4. Van Megen, H.; van den Elsen, S.; Holterman, M.; Karssen, G.; Mooyman, P.; Bongers, T.; Holovachov, O.; Bakker, J.; Helder, J. A phylogenetic tree of nematodes based on about 1200 full-length small subunit ribosomal DNA sequences. *Nematology* **2009**, *11*, 927–950.
5. Schwarz, E.M.; Hu, Y.; Antoshechkin, I.; Miller, M.M.; Sternberg, P.W.; Aroian, R.V. The genome and transcriptome of the zoonotic hookworm *Ancylostoma ceylanicum* identify infection-specific gene families. *Nat. Genet.* **2015**, *47*, 416–422.
6. Abubucker, S.; Martin, J.; Yin, Y.; Fulton, L.; Yang, S.P.; Hallsworth-Pepin, K.; Johnston, J.S.; Hawdon, J.; McCarter, J.P.; Wilson, R.K.; et al. The canine hookworm genome: Analysis and classification of *Ancylostoma caninum* survey sequences. *Mol. Biochem. Parasitol.* **2008**, *157*, 187–192.
7. Howe, K.L.; Bolt, B.J.; Cain, S.; Chan, J.; Chen, W.J.; Davis, P.; Done, J.; Down, T.; Gao, S.; Grove, C.; et al. WormBase 2016: Expanding to enable helminth genomic research. *Nucleic Acids Res.* **2016**, *44*, D774–D780.
8. Mortazavi, A.; Schwarz, E.M.; Williams, B.; Schaeffer, L.; Antoshechkin, I.; Wold, B.J.; Sternberg, P.W. Scaffolding a *Caenorhabditis* nematode genome with RNA-seq. *Genome Res.* **2010**, *20*, 1740–1747.
9. Stein, L.D.; Bao, Z.; Blasiar, D.; Blumenthal, T.; Brent, M.R.; Chen, N.; Chinwalla, A.; Clarke, L.; Clee, C.; Coghlan, A.; et al. The genome sequence of *Caenorhabditis briggsae*: A platform for comparative genomics. *PLoS Biol.* **2003**, *1*, e45.
10. Koutsovoulos, G.; Makepeace, B.; Tanya, V.N.; Blaxter, M. Palaeosymbiosis revealed by genomic fossils of *Wolbachia* in a strongyloidean nematode. *PLoS Genet.* **2014**, *10*, e1004397.
11. Godel, C.; Kumar, S.; Koutsovoulos, G.; Ludin, P.; Nilsson, D.; Comandatore, F.; Wrobel, N.; Thompson, M.; Schmid, C.D.; Goto, S.; et al. The genome of the heartworm, *Dirofilaria immitis*, reveals drug and vaccine targets. *FASEB J.* **2012**, *26*, 4650–4661.
12. Laing, R.; Kikuchi, T.; Martinelli, A.; Tsai, I.J.; Beech, R.N.; Redman, E.; Holroyd, N.; Bartley, D.J.; Beasley, H.; Britton, C.; et al. The genome and transcriptome of *Haemonchus contortus*, a key model parasite for drug and vaccine discovery. *Genome Biol.* **2013**, *14*, R88, doi:10.1186/gb-2013-14-8-r88.
13. Bai, X.; Adams, B.J.; Ciche, T.A.; Clifton, S.; Gaugler, R.; Kim, K.S.; Spieth, J.; Sternberg, P.W.; Wilson, R.K.; Grewal, P.S. A lover and a fighter: The genome sequence of an entomopathogenic nematode *Heterorhabditis bacteriophora*. *PLoS ONE* **2013**, *8*, e69618.
14. Tallon, L.J.; Liu, X.; Bennuru, S.; Chibucos, M.C.; Godinez, A.; Ott, S.; Zhao, X.; Sadzewicz, L.; Fraser, C.M.; Nutman, T.B.; et al. Single molecule sequencing and genome assembly of a clinical specimen of *Loa loa*, the causative agent of loiasis. *BMC Genom.* **2014**, *15*, 788, doi:10.1186/1471-2164-15-788.
15. Tang, Y.T.; Gao, X.; Rosa, B.A.; Abubucker, S.; Hallsworth-Pepin, K.; Martin, J.; Tyagi, R.; Heizer, E.; Zhang, X.; Bhonagiri-Palsikar, V.; et al. Genome of the human hookworm *Necator americanus*. *Nat. Genet.* **2014**, *46*, 261–269.
16. Srinivasan, J.; Dillman, A.R.; Macchietto, M.G.; Heikkinen, L.; Lakso, M.; Fracchia, K.M.; Antoshechkin, I.; Mortazavi, A.; Wong, G.; Sternberg, P.W. The draft genome and transcriptome of *Panagrellus redivivus* are shaped by the harsh demands of a free-living lifestyle. *Genetics* **2013**, *193*, 1279–1295.
17. Dieterich, C.; Clifton, S.W.; Schuster, L.N.; Chinwalla, A.; Delehaunty, K.; Dinkelacker, I.; Fulton, L.; Fulton, R.; Godfrey, J.; Minx, P.; et al. The *Pristionchus pacificus* genome provides a unique perspective on nematode lifestyle and parasitism. *Nat. Genet.* **2008**, *40*, 1193–1198.

18. Schiffer, P.H.; Kroiher, M.; Kraus, C.; Koutsovoulos, G.D.; Kumar, S.; Camps, J.I.; Nsah, N.A.; Stappert, D.; Morris, K.; Heger, P.; et al. The genome of *Romanomermis culicivorax*: Revealing fundamental changes in the core developmental genetic toolkit in Nematoda. *BMC Genom.* **2013**, *14*, 923, doi:10.1186/1471-2164-14-923.
19. Dillman, A.R.; Macchietto, M.; Porter, C.F.; Rogers, A.; Williams, B.; Antoshechkin, I.; Lee, M.M.; Goodwin, Z.; Lu, X.; Lewis, E.E.; et al. Comparative genomics of *Steinernema* reveals deeply conserved gene regulatory networks. *Genome Biol.* **2015**, *16*, 200, doi:10.1186/s13059-015-0746-6.
20. Hunt, V.L.; Tsai, I.J.; Coghlan, A.; Reid, A.J.; Holroyd, N.; Foth, B.J.; Tracey, A.; Cotton, J.A.; Stanley, E.J.; Beasley, H.; et al. The genomic basis of parasitism in the *Strongyloides* clade of nematodes. *Nat. Genet.* **2016**, *48*, 299–307.
21. Mitreva, M.; Jasmer, D.P.; Zarlenga, D.S.; Wang, Z.; Abubucker, S.; Martin, J.; Taylor, C.M.; Yin, Y.; Fulton, L.; Minx, P.; et al. The draft genome of the parasitic nematode *Trichinella spiralis*. *Nat. Genet.* **2011**, *43*, 228–235.
22. Foth, B.J.; Tsai, I.J.; Reid, A.J.; Bancroft, A.J.; Nichol, S.; Tracey, A.; Holroyd, N.; Cotton, J.A.; Stanley, E.J.; Zarowiecki, M.; et al. Whipworm genome and dual-species transcriptome analyses provide molecular insights into an intimate host-parasite interaction. *Nat. Genet.* **2014**, *46*, 693–700.
23. Sequencing Consortium. Genome sequence of the nematode *C. elegans*: A platform for investigating biology. *Science* **1998**, *282*, 2012–2018.
24. Ghedin, E.; Wang, S.; Spiro, D.; Caler, E.; Zhao, Q.; Crabtree, J.; Allen, J.E.; Delcher, A.L.; Guiliano, D.B.; Miranda-Saavedra, D.; et al. Draft genome of the filarial nematode parasite *Brugia malayi*. *Science* **2007**, *317*, 1756–1760.
25. Jex, A.R.; Liu, S.; Li, B.; Young, N.D.; Hall, R.S.; Li, Y.; Yang, L.; Zeng, N.; Xu, X.; Xiong, Z.; et al. *Ascaris suum* draft genome. *Nature* **2011**, *479*, 529–533.
26. Cotton, J.A.; Lilley, C.J.; Jones, L.M.; Kikuchi, T.; Reid, A.J.; Thorpe, P.; Tsai, I.J.; Beasley, H.; Blok, V.; Cock, P.J.; et al. The genome and life-stage specific transcriptomes of *Globodera pallida* elucidate key aspects of plant parasitism by a cyst nematode. *Genome Biol.* **2014**, *15*, R43, doi:10.1186/gb-2014-15-3-r43.
27. Opperman, C.H.; Bird, D.M.; Williamson, V.M.; Rokhsar, D.S.; Burke, M.; Cohn, J.; Cromer, J.; Diener, S.; Gajan, J.; Graham, S.; et al. Sequence and genetic map of *Meloidogyne hapla*: A compact nematode genome for plant parasitism. *Proc. Natl. Acad. Sci. USA* **2008**, *105*, 14802–14807.
28. Abad, P.; Gouzy, J.; Aury, J.M.; Castagnone-Sereno, P.; Danchin, E.G.; Deleury, E.; Perfus-Barbeoch, L.; Anthouard, V.; Artiguenave, F.; Blok, V.C.; et al. Genome sequence of the metazoan plant-parasitic nematode *Meloidogyne incognita*. *Nat. Biotechnol.* **2008**, *26*, 909–915.
29. Kikuchi, T.; Cotton, J.A.; Dalzell, J.J.; Hasegawa, K.; Kanzaki, N.; McVeigh, P.; Takanashi, T.; Tsai, I.J.; Assefa, S.A.; Cock, P.J.; et al. Genomic insights into the origin of parasitism in the emerging plant pathogen *Bursaphelenchus xylophilus*. *PLoS Pathog.* **2011**, *7*, e1002219.
